# The prevalence of chronic and episodic loneliness and social isolation from a longitudinal survey

**DOI:** 10.1038/s41598-023-39289-x

**Published:** 2023-08-01

**Authors:** Michelle H. Lim, Karine E. Manera, Katherine B. Owen, Philayrath Phongsavan, Ben J. Smith

**Affiliations:** 1 Sydney School of Public Health, Faculty of Medicine and Health, The University of Sydney, Sydney, NSW Australia; 2 Charles Perkins Centre, The University of Sydney, Sydney, NSW Australia; 3 Iverson Health Innovation Research Institute, Swinburne University of Technology, Victoria, Australia

**Keywords:** Psychology, Risk factors

## Abstract

Loneliness and social isolation, experienced more long-term, has been shown to increase mortality and lead to poorer health outcomes in specific cohorts. However, it is unclear what the prevalence of chronic loneliness and social isolation is, and which demographic groups are most at risk of reporting more chronic forms. A psychometrically validated classification system was used to identify people who met criteria for episodic and chronic loneliness and social isolation using the Household Income and Labour Dynamics in Australia (HILDA) survey waves 14–18. The prevalence of loneliness (overall 34%; 21% episodic, 13% chronic) far exceeded that of social isolation (overall 17%; 13% episodic, 4% chronic). There was consistency in the demographic characteristics (from age, sex, household type, income) of those who experienced loneliness and social isolation. However, people with a long-term health condition had an elevated risk of episodic loneliness (AOR 1.24, 95% CI 1.11–1.39) and a markedly higher risk of chronic loneliness (AOR 2.01, 95% CI 1.76–2.29), compared with those without a long-term health condition. Loneliness, both episodic and chronic subtypes, is more prevalent than social isolation. However, both chronic loneliness and social isolation remains neglected and poorly targeted within current practice and policy.

## Introduction

Being socially connected is fundamental to human health and wellbeing^1^. For decades, social isolation, characterised by an objective lack of social contact and connections^2,3^, has been widely acknowledged as a risk factor for broad-based mortality and morbidity^4,5^. But there has been a growing number of studies that have found similar harmful effects of loneliness on health^6^. Loneliness—sometimes described as ‘perceived social isolation’, is characterised by distressing feelings when one perceives a discrepancy between desired and actual relationships^7^. Loneliness is regarded as a biopsychosocial stressor^8,9^, and associated with a multitude of poor health outcomes, from cardiometabolic disease, stroke^10^, dementia^11^, and depression^12^. It is therefore not just having social connections but also reporting *feeling* socially connected to others, that holds implications for one’s risk of morbidity and mortality^13,14^.

Both social isolation and loneliness share similar associations with demographic, socio-economic and health factors^5,15^. Socio-demographic characteristics such as age, gender, living alone, socioeconomic status, migrant status and employment are all factors that have been examined in both the social isolation and loneliness research^3^. Older adults are often perceived to be more vulnerable to loneliness and social isolation as compared with other age groups^16^. However, it is now widely accepted that loneliness and social isolation affects everyone across the life course^17^.

The impacts of social isolation and loneliness on health are further influenced by a broader number of factors including the social determinants of health in which people may be born into or live under^18^. For example, in studies on social isolation, factors including neighbourhood disadvantage and migrant status play a role in moderating poorer health outcomes including myocardial infarction risk^19^, colorectal cancer risk^20^, carotid stiffness^21^, and psychological wellbeing^22^. Current models of loneliness acknowledge the influence of the social environment and resources available to the lonely individual^15,23,24^, and it has been reported that some individuals are more likely to break out of the cycle of loneliness than others^25^. Indeed, poor health, be it physical or mental^26^, influences one’s capacity to participate socially in their networks including friends, colleagues, and the wider community. Both loneliness and social isolation are linked to higher incidence of chronic disease^27,28^.

Population surveys show varying prevalence rates of loneliness and social isolation across different samples and stages. The reported global prevalence rate in a recent meta-analysis noted that the prevalence of loneliness in adolescents (12–17 years old) differed depending on the region (9.2% South-East Asian to 14.4% Eastern Mediterranean countries) and for young people (18–29 years old) was 5.3%^29^. Middle aged adults (30–59 years old) reported a pooled prevalence estimate of 6.9% across different parts of European, and for older adults (over 60 years) reported anywhere from 5.2% (Northern Europe), 8.7% (Western Europe), 15.7% Southern European to the highest 21.3% for Eastern Europeans countries^29^.

While there is robust scientific literature on the negative impact of loneliness and social isolation on health, there has been far less scrutiny on the prevalence rates, sociodemographic risk factors, and the health impacts of persistent experiences (i.e., chronic) loneliness and social isolation. Specifically, social isolation is well-known to be detrimental for health and wellbeing^4,5^ but the evidence on chronic social isolation is largely informed by numerous animal studies (due to ethical reasons)^6^ or retrospective epidemiological data^30^. The study of chronic loneliness on the other hand, remains underexamined because most of this work has been reliant on cross-sectional study designs which may not capture the persistence or stability of these experiences^31^. Many loneliness measures, both direct, such as Office of National Statistics Loneliness item^32^, and indirect measures UCLA-Loneliness Scale^33^ and De Jong Gierveld Social and Emotional Loneliness scale^34^ often capture loneliness through the frequency of lonely feelings (i.e., *how often do you feel isolated from others?*). This is different to the capturing the persistence of lonely feelings (i.e., *how long have you felt isolated from others?*) or intensity (e.g., how distressed did you feel?). Attention to the cumulative impact of more chronic experiences of loneliness on poorer health outcomes is growing^35^ and examined within particular cohorts including older adults^36–38^ and adolescents^39^. For instance, chronic loneliness has been found to be associated with an 80% greater risk of death in Americans aged 50 and older (situational HR 1.56%; 95% CI 1.52–1.62 versus chronic HR 1.83, 95% CI 1.71–1.87)^37^.

## Study aims

This study will add to the growing literature on how chronic forms of loneliness and social isolation affects different demographic groups. First, we aimed to identify the proportion of people who experience loneliness and social isolation persistently over time (i.e., chronicity), as opposed to transient periods (i.e., episodic). Second, we aimed to identify socio-demographic characteristics that were associated with episodic or chronic loneliness and social isolation.

## Methods

### Participants and data source

Data used in this study were collected from the Household Income and Labour Dynamics in Australia (HILDA) survey a household-based panel study of over 9,000 Australian households conducted annually, with wave 1 starting in 2001^40^. A number of household-level and person-level questionnaires were completed for each household. This study uses data from the self-completion questionnaire, which is undertaken by individuals aged 15 years or older in each participating household, from waves 14–18 (5 years). We included cases that did not have missing data for loneliness and social isolation items across all waves. In total, we had 10,746 participants from waves 14–18 with no missing loneliness items, and 10,918 participants with no missing social isolation items from waves 14–18.

### Measures

The HILDA survey collects data on household and family relationships, income, education, employment, living situation, lifestyle behaviours and general health and wellbeing.

#### Loneliness and social isolation

Previous estimates of the prevalence of loneliness among HILDA participants have been based on responses to the single-item statement “I often feel very lonely”^41^. Using a single item may however lead to underreporting due to the known stigma of loneliness^42^. To assess loneliness and social isolation, we used the scales derived from 10-items Index of Social Support scale^43^ in the HILDA questionnaire (see Supplementary Fig. 1 in psychometric validation paper)^44^. The loneliness scale included three items: “People don’t come to visit me as often as I would like”, “I often need help from other people but can’t get it” and “I often feel very lonely”. The social isolation scale included four items: “There is someone who can always cheer me up when I’m down”, “I enjoy the time I spend with the people who are important to me”, “When something’s on my mind, just talking with the people I know can make me feel better” and “When I need someone to help me out, I can usually find someone”. Psychometric analysis has shown that each of these scales has good internal reliability and construct validity^44^. We classified people who were lonely to have a median score of greater than 4, and for social isolation a median item score of less than 4, on the scales developed for each of these conditions (see methodology in psychometric validation paper)^44^.

To date, the study of the impact of chronic loneliness and social isolation is in its infancy. In this study, we defined episodic loneliness and social isolation as meeting criteria for any episode or number of episodes that were not consecutive (e.g., only wave 15, or both waves 15 and 17). Chronic loneliness and social isolation were defined as meeting criteria for two or more consecutive episodes (e.g., waves 14 and 15, or waves 16, 17, and 18); in other words, a minimum of two consecutive years. Episodic and chronic loneliness, and episodic and chronic social isolation were mutually exclusive, and each person was categorised as have episodic, chronic, or no loneliness, and episodic, chronic, or no social isolation across the five-year period (waves 14–18).

The term ‘chronic’ is therefore used to indicate that the experience is prolonged or persistent over a longer period of time and may be resistant to change. This is consistent with American Psychological Association’s definition of chronicity (see https://dictionary.apa.org/chronic). While loneliness and social isolation are social experiences and not diseases, our classification of what is chronic is also aligned to Center for Disease Control’s definition of chronic disease^45^.

#### Socio-demographic characteristics

We identified nine socio-demographic characteristics known to be associated with loneliness, social isolation, or both based on previous research.

For sex only two categories were used: identifying as male or female. Age was categorised into five groups: 15–29 years; 30–44 years; 45–59 years; 60–74 years; or 75 years and over. Loneliness and social isolation are known to vary across the lifespan, and this can also vary across sex^17,31,46^, hence examining the impact of loneliness and social isolation across both sex and age is critical.

Two socio-demographic characteristics that could influence the degree of social interaction one may have include household structure (i.e., living status) and employment. Living alone and unemployment have been shown to contribute to loneliness^47–49^ and social isolation^50^ in different populations. Household structure was categorised as follows: (1) couple with child; (2) couple without child; (3) lone parent with child; (4) lone person; or (5) other, which included unrelated household members, other family members, and single or double-parent households with a child over the age of 15. The employment status of participants was classified as: (1) full-time; (2) part-time; (3) retired; (4) unemployed; or 5) other (which included home duties, non-working students and unspecified).

An established social identity^51^ and ability to communicate in the primary language in the country one lives in^52^ were identified as socio-demographic characteristics that could influence social integration. Indeed, being of ethnic minority status is increasingly being examined as an increased risk for loneliness^49^ and social isolation^53^. Because migrant status was not directly available, we selected country of birth and language spoke at home. Country of birth was categorised as: (1) Australia; (2) Other English-speaking country; or (3) Other non-English speaking country. Language spoken at home was categorised as (1) English or (2) other.

Two socio-demographic characteristics were selected to indicate economic status, which were household income and neighbourhood disadvantage, both well known to influence loneliness and social isolation. More generally, lower household income and increased neighbourhood disadvantage is associated with increased risk for loneliness and social isolation^48,54^. Household income was categorised as three levels: (1) less than $80,000; (2) $80,000–$149,999; or (3) more than $150,000. In order to determine relative neighbourhood disadvantage, the composite Socio-Economic Index for Areas and Index of Relative Socio-Economic Advantage and Disadvantage (SEIFA IRSAD) was used. This captures several variables including income, education, employment, occupation, and housing characteristics at the postcode level, based on the Australian Population Census^55^. Participants were classified into a SEIFA IRSAD quintile, based on their postcode of residence, with quintile 5 indicating the highest levels of socio-economic advantage and quintile 1 the lowest levels of socio-economic advantage.

We also included a measure of the presence of long-term health conditions (i.e., chronic disease) to examine how this relates to both loneliness and social isolation. In large epidemiological studies, loneliness and social isolation have been found to be associated with a higher incidence of chronic disease^10,28,56^. This categorical variable (Yes/No) was coded based on self-reporting of at least one of many listed physical health conditions (e.g., heart disease, arthritis, chronic pain, hearing, or speech problems).

### Data analysis

In order to derive prevalence estimates of loneliness and social isolation, the longitudinal weights provided by HILDA were used, which adjust for attrition and ensure that the sample is representative of the Australian population distribution of age, sex, state, labour force status, marital status, and household composition. A detailed description of the HILDA survey weights is available elsewhere^57^. First, we estimated the prevalence of episodic, chronic or any loneliness and social isolation across socio-demographic characteristics and across the HILDA waves 14 (2014) to 18 (2018). Second, we conducted univariable and multivariable logistic regression to assess the difference in episodic, chronic, or either subtype (episodic or chronic) of loneliness and isolation based on the specified socio-demographic characteristics. See Table 1 for episodic and chronic loneliness and Table 2 for episodic and chronic social isolation.

**Table 1:** Episodic and chronic loneliness by participant characteristics, *n* (%) [weighted%].

	Episodic (n = 2222)	Crude OR (95% CI)	AOR^a^ (95% CI)	Chronic (n = 1458)	OR (95% CI)	AOR^a^ (95% CI)	Any loneliness (n = 3680)	OR (95% CI)	AOR^a^ (95% CI)
Sex									
Female	1236 (21) [22]	Ref	Ref	819 (14) [13]	Ref	Ref	2055 (35) [35]	Ref	Ref
Male	986 (20) [20]	**0.91 (0.83–1.00)**	**0.96 (0.87–1.06)**	639 (13) [13]	**0.90 (0.80–1.00)**	**0.97 (0.86–1.10)**	1625 (32) [33]	0.89 (0.82–0.96)**	**0.96 (0.88–1.05)**
Age group									
15–29	362 (21) [22]	**1.04 (0.90–1.21)**	**1.02 (0.86–1.21)**	217 (13) [12]	**0.87 (0.73–1.04)**	0.77 (0.62–0.94)**	579 (34) [35]	**0.96 (0.84–1.09)**	**0.87 (0.75–0.97)**
30–44	541 (21) [22]	Ref	Ref	379 (14) [14]	Ref	Ref	920 (35) [35]	Ref	Ref
45–59	572 (19) [19]	**0.90 (0.79–1.03)**	**0.89 (0.77–1.03)**	436 (14) [14]	**1.00 (0.86–1.16)**	**0.88 (0.74–1.04)**	1008 (33) [33]	**0.93 (0.83–1.04)**	0.85 (0.75–0.97)*
60–74	529 (21) [20]	**1.02 (0.89–1.16)**	**0.95 (0.78–1.15)**	290 (11) [12]	0.77 (0.65–0.90)**	0.62 (0.49–0.78)***	819 (32) [32]	0.89 (0.79–0.99)*	0.74 (0.62–0.88)***
75 +	218 (22) [21]	**1.08 (0.90–1.28)**	**0.89 (0.69–1.15)**	136 (14) [13]	**0.93 (0.76–1.15)**	0.59 (0.43–0.81)**	354 (35) [34]	**1.02 (0.87–1.18)**	0.67 (0.54–0.85)***
Household structure									
Couple w child	510 (20) [21]	Ref	Ref	316 (12) [10]	Ref	Ref	826 (32) [31]	Ref	Ref
Couple wo child	677 (18) [18]	**0.92 (0.81–1.05)**	**0.87 (0.74–1.01)**	330 (9) [9]	0.71 (0.61–0.84)***	0.75 (0.62–0.92)**	1007 (27) [27]	0.81 (0.73–0.90)***	0.80 (0.70–0.92)**
Lone parent w child	76 (29) [30]	1.64 (1.24–2.18)***	**1.21 (0.89–1.63)**	72 (27) [29]	2.69 (2.00–3.61)***	1.64 (1.19–2.27)**	148 (55) [59]	2.69 (2.09–3.47)***	1.68 (1.28–2.21)***
Lone person	473 (25) [26]	1.40 (1.21–1.61)***	**1.14 (0.96–1.36)**	400 (21) [22]	1.98 (1.69–2.33)***	1.66 (1.36–2.03)***	873 (47) [47]	1.90 (1.68–2.15)***	1.52 (1.30–1.77)***
Other	486 (20) [21]	**1.03 (0.89–1.18)**	**1.01 (0.86–1.19)**	340 (14) [15]	**1.18 (1.00–1.39)**	**1.20 (0.99–1.45)**	826 (34) [36]	**1.11 (0.98–1.24)**	**1.12 (0.98–1.29)**
Country of birth^b^									
Australia	1729 (20) [20]	Ref	Ref	1178 (14) [14]	Ref	Ref	2907 (34) [34]	Ref	Ref
Other English speaking	216 (20) [21]	**0.97 (0.83–1.14)**	**1.01 (0.86–1.19)**	139 (13) [13]	**0.91 (0.76–1.10)**	**1.03 (0.85–1.26)**	355 (32) [33]	**0.94 (0.82–1.07)**	**1.02 (0.88–1.18)**
Other non-English speaking	276 (23) [25]	1.18 (1.02–1.37)*	1.22 (1.01–1.48)*	140 (12) [10]	0.83 (0.69–1.00)*	**0.82 (0.64–1.05)**	416 (35) [35]	**1.03 (0.91–1.17)**	**1.06 (0.89–1.26)**
Speak language other than English									
No	2000 (20) [20]	Ref	Ref	1331 (14) [14]	Ref	Ref	3331 (34) [34]	Ref	Ref
Yes	222 (22) [24]	**1.11 (0.95–1.30)**	**0.98 (0.79–1.20)**	127 (13) [11]	**0.92 (0.76–1.12)**	**1.06 (0.82–1.38)**	349 (35) [34]	**1.04 (0.90–1.19)**	**1.00 (0.83–1.21)**
Household income^b^									
150,000 +	427 (15) [16]	Ref	Ref	239 (9) [9]	Ref	Ref	666 (24) [24]	Ref	Ref
80,000–149,999	680 (20) [21]	1.36 (1.19–1.55)***	1.27 (1.10–1.45)***	399 (12) [12]	1.40 (1.18–1.66)***	1.26 (1.05–1.50)*	1079 (31) [33]	1.45 (1.30–1.63)***	1.31 (1.16–1.47)***
< 80,000	1093 (24) [25]	1.80 (1.59–2.04)***	1.49 (1.27–1.74)***	801 (18) [18]	2.34 (2.01–2.73)***	1.66 (1.36–2.02)***	1894 (42) [42]	2.36 (2.13–2.62)***	1.73 (1.51–1.98)***
Employment status^b^									
Full time	872 (19) [19]	Ref	Ref	530 (11) [11]	Ref	Ref	1402 (30) [30]	Ref	Ref
Part time	438 (19) [20]	**1.05 (0.92–1.19)**	**0.96 (0.84–1.10)**	276 (12) [12]	**1.09 (0.93–1.27)**	**0.97 (0.82–1.15)**	714 (31) [32]	**1.08 (0.97–1.20)**	**0.96 (0.85–1.08)**
Retired	558 (21) [21]	1.19 (1.06–1.34)**	**0.94 (0.77–1.14)**	328 (13) [12]	**1.13 (0.97–1.30)**	**0.92 (0.72–1.16)**	886 (34) [33]	1.20 (1.09–1.33)***	**0.90 (0.76–1.07)**
Unemployed	79 (29) [29]	1.76 (1.34–2.31)***	1.33 (1.01–1.77)*	70 (25) [25]	2.67 (2.01–3.56)***	1.66 (1.22–2.25)**	149 (54) [54]	2.76 (2.16–3.53)***	1.80 (1.39–2.33)***
Other^c^	274 (27) [28]	1.61 (1.38–1.89)***	1.21 (1.02–1.44)*	253 (25) [26]	2.60 (2.19–3.07)***	1.57 (1.29–1.91)***	527 (52) [54]	2.52 (2.19–2.89)***	1.60 (1.37–1.87)***
SEIFA IRSAD quintile									
5	409 (17) [18]	Ref	Ref	244 (10) [11]	Ref	Ref	653 (28) [30]	Ref	Ref
4	405 (18) [18]	**1.03 (0.89–1.20)**	**0.99 (0.85–1.15)**	231 (10) [10]	**0.98 (0.81–1.18)**	**0.89 (0.73–1.08)**	636 (28) [27]	**1.01 (0.89–1.15)**	**0.94 (0.82–1.07)**
3	419 (20) [21]	1.17 (1.01–1.36)*	**1.08 (0.92–1.26)**	282 (13) [14]	1.33 (1.11–1.59)**	**1.13 (0.93–1.37)**	701 (33) [35]	1.29 (1.13–1.46)***	**1.12 (0.98–1.28)**
2	474 (22) [22]	1.33 (1.15–1.54)***	**1.15 (0.98–1.34)**	329 (15) [15]	1.55 (1.30–1.85)***	**1.19 (0.98–1.44)**	803 (37) [37]	1.53 (1.35–1.74)***	1.21 (1.06–1.38)**
1	515 (26) [27]	1.69 (1.46–1.95)***	1.36 (1.16–1.59)***	372 (19) [17]	2.02 (1.69–2.40)***	1.34 (1.11–1.62)**	887 (45) [44]	2.14 (1.88–2.43)***	1.50 (1.31–1.73)***
Long term health condition^b^									
No	1415 (19) [20]	Ref	Ref	784 (10) [10]	Ref	Ref	2199 (29) [30]	Ref	Ref
Yes	806 (24) [24]	1.40 (1.27–1.54)***	1.24 (1.11–1.39)***	673 (20) [21]	2.21 (1.97–2.47)***	2.01 (1.76–2.29)***	1479 (45) [44]	1.97 (1.81–2.14)***	1.75 (1.59–1.93)***

Significant values are in bold.

*SEIFA* Socio-Economic Indexes for Areas, *IRSAD* Index of Relative Socio-economic Advantage and Disadvantage, with quintile 5 indicating the highest levels of socio-economic advantage and quintile 1 the lowest levels of socio-economic advantage. *p* values included where significant.

*p < 0.05; **p < 0.01; ***p < 0.001, ^a^n = 10,738. Significant results are represented in black font. All variables included in the model, ^b^Missing data from ‘don’t know’, ‘refused’ or ‘unable to determine value’, ^c^Other includes home duties, non-working students and other (unspecified).

**Table 2:** Episodic and chronic social isolation by participant characteristics, *n* (%) [weighted%].

	Episodic (n = 1423)	OR (95% CI)	AOR^a^ (95% CI)	Chronic (n = 432)	OR (95% CI)	AOR^a^ (95% CI)	Any social isolation (n = 1855)	OR (95% CI)	AOR^a^ (95% CI)
Sex									
Female	725 (12)[13]	Ref	Ref	188 (3) [4]	Ref	Ref	913 (15) [17]	Ref	Ref
Male	698 (14) [15]	1.16 (1.04–1.30)**	1.23 (1.09–1.38)***	244 (5) [5]	1.56 (1.29–1.89)***	1.78 (1.45–2.20)***	942 (19) [20]	1.27 (1.15–1.40)***	1.39 (1.25–1.55)***
Age group									
15–29	213 (13) [12]	1.22 (1.01–1.48)*	**1.02 (0.83–1.27)**	68 (4) [5]	**1.26 (0.91–1.74)**	**0.99 (0.69–1.41)**	281 (17) [18]	1.25 (1.05–1.48)*	**1.00 (0.83–1.21)**
30–44	278 (11) [13]	Ref	Ref	85 (3) [4]	Ref	Ref	363 (14) [17]	Ref	Ref
45–59	416 (14) [14]	1.35 (1.15–1.59)***	**1.16 (0.97–1.39)**	150 (5) [6]	1.56 (1.19–2.05)**	**1.23 (0.91–1.66)**	566 (19) [20]	1.44 (1.24–1.66)***	1.19 (1.01–1.40)*
60–74	364 (14) [15]	1.42 (1.20–1.67)***	**1.00 (0.79–1.27)**	89 (4) [4]	**1.09 (0.80–1.47)**	**0.75 (0.49–1.13)**	453 (18) [18]	1.36 (1.17–1.58)***	**0.91 (0.73–1.13)**
75 +	152 (15) [14]	1.47 (1.19–1.81)***	**0.87 (0.64–1.18)**	40 (4) [4]	**1.21 (0.83–1.78)**	**0.65 (0.38–1.12)**	192 (19) [19]	1.43 (1.18–1.74)***	**0.77 (0.59–1.02)**
Household structure									
Couple w child	251 (10) [11]	Ref	Ref	70 (3) [4]	Ref	Ref	321 (12) [15]	Ref	Ref
Couple wo child	466 (13) [12]	1.35 (1.15–1.59)***	**1.11 (0.91–1.35)**	102 (3) [4]	**1.03 (0.76–1.40)**	**0.98 (0.68–1.40)**	568 (15) [15]	1.29 (1.12–1.50)***	**1.10 (0.92–1.31)**
Lone parent w child	49 (18) [19]	2.11 (1.51–2.95)***	1.50 (1.05–2.15)*	17 (6) [9]	2.46 (1.43–4.25)**	**1.62 (0.89–2.96)**	66 (25) [27]	2.34 (1.73–3.16)***	1.58 (1.14–2.19)**
Lone person	298 (16) [16]	1.78 (1.49–2.13)***	1.26 (1.02–1.56)*	117 (6) [7]	2.42 (1.79–3.28)***	1.82 (1.28–2.61)***	415 (22) [23]	2.03 (1.73–2.39)***	1.45 (1.19–1.76)***
Other	359 (15) [16]	1.62 (1.36–1.92)***	1.44 (1.18–1.75)***	126 (5) [6]	1.97 (1.46–2.65)***	1.72 (1.23–2.41)**	485 (20) [21]	1.76 (1.51–2.05)***	1.57 (1.32–1.88)***
Country of birth^b^									
Australia	1123 (13) [13]	Ref	Ref	333 (4) [4]	Ref	Ref	1456 (17) [18]	Ref	Ref
Other English speaking	157 (14) [14]	**1.12 (0.94–1.34)**	**1.12 (0.93–1.35)**	39 (4) [4]	**0.92 (0.66–1.29)**	**1.01 (0.71–1.43)**	196 (18) [18]	**1.08 (0.91–1.27)**	**1.10 (0.93–1.31)**
Other non–English speaking	141 (12) [15]	**0.89 (0.74–1.07)**	**0.85 (0.66–1.09)**	60 (5) [7]	**1.31 (0.99–1.74)**	**1.28 (0.87–1.88)**	201 (17) [22]	**0.99 (0.84–1.17)**	**0.95 (0.76–1.18)**
Speak language other than English									
No	1300 (13) [13]	Ref	Ref	380 (4) [4]	Ref	Ref	1680 (17) [18]	Ref	Ref
Yes	123 (12) [16]	**0.92 (0.75–1.12)**	**1.03 (0.79–1.34)**	52 (5) [7]	1.36 (1.01–1.83)*	**1.28 (0.85–1.92)**	175 (17) [22]	**1.03 (0.87–1.22)**	**1.10 (0.87–1.39)**
Household income^b^									
150,000 +	251 (9) [10]	Ref	Ref	66 (2) [2]	Ref	Ref	317 (11) [12]	Ref	Ref
80,000–149,999	407 (12) [13]	1.36 (1.15–1.61)***	1.23 (1.04–1.47)*	106 (3) [4]	**1.32 (0.96–1.80)**	**1.16 (0.84–1.60)**	513 (15) [17]	1.37 (1.18–1.59)***	1.23 (1.05–1.44)**
< 80,000	748 (17) [17]	2.03 (1.74–2.36)***	1.48 (1.22–1.80)***	252 (6) [7]	2.46 (1.87–3.24)***	1.83 (1.30–2.57)***	1000 (22) [24]	2.24 (1.96–2.57)***	1.62 (1.36–1.93)***
Employment status^b^									
Full time	517 (11) [12]	Ref	Ref	155 (3) [4]	Ref	Ref	672 (14) [15]	Ref	Ref
Part time	246 (11) [11]	**0.99 (0.84–1.16)**	**0.95 (0.80–1.13)**	71 (3) [5]	**0.95 (0.72–1.27)**	**1.03 (0.76–1.40)**	317 (14) [16]	**0.98 (0.85–1.13)**	**0.97 (0.83–1.14)**
Retired	409 (15) [16]	1.47 (1.28–1.69)***	**1.17 (0.93–1.46)**	102 (4) [4]	**1.17 (0.91–1.51)**	**1.02 (0.68–1.53)**	511 (19) [20]	1.43 (1.26–1.62)***	**1.15 (0.93–1.41)**
Unemployed	56 (20) [27]	2.07 (1.52–2.82)***	1.43 (1.03–1.97)*	24 (9) [9]	2.81 (1.79–4.40)***	1.66 (1.03–2.67)*	80 (29) [36]	2.46 (1.88–3.24)***	1.60 (1.20–2.14)**
Other^c^	193 (19) [20]	1.92 (1.60–2.30)***	1.47 (1.19–1.80)***	79 (8) [9]	2.49 (1.88–3.30)***	1.83 (1.32–2.53)***	272 (27) [29]	2.21 (1.88–2.60)***	1.67 (1.39–2.01)***
SEIFA IRSAD quintile									
5	240 (10) [12]	Ref	Ref	44 (2) [3]	Ref	Ref	284 (12) [15]	Ref	Ref
4	229 (10) [11]	**0.99 (0.82–1.19)**	**0.92 (0.75–1.11)**	95 (4) [5]	2.29 (1.59–3.29)***	2.11 (1.46–3.06)***	324 (14) [16]	1.21 (1.02–1.44)*	**1.12 (0.94–1.33)**
3	260 (12) [12]	1.23 (1.02–1.48)*	**1.12 (0.92–1.35)**	104 (5) [6]	2.70 (1.89–3.96)***	2.33 (1.61–3.38)***	364 (17) [18]	1.50 (1.27–1.78)***	1.34 (1.12–1.59)
2	314 (14) [16]	1.49 (1.25–1.79)***	1.24 (1.03–1.50)*	94 (4) [5]	2.38 (1.66–3.42)***	1.77 (1.21–2.59)**	408 (19) [20]	1.69 (1.43–1.99)***	1.35 (1.14–1.61)**
1	380 (19) [20]	2.12 (1.78–2.53)***	1.63 (1.35–1.97)***	95 (5) [6]	2.68 (1.87–3.85)***	1.69 (1.15–2.48)**	475 (24) [25]	2.34 (1.99–2.75)***	1.68 (1.41–2.00)***
Long term health condition^b^									
No	839 (11) [12]	Ref	Ref	224 (3) [4]	Ref	Ref	1063 (14) [16]	Ref	Ref
Yes	584 (18) [18]	1.71 (1.52–1.91)***	1.34 (1.17–1.52)***	208 (6) [7]	2.18 (1.80–2.65)***	1.87 (1.50–2.34)***	792 (24) [25]	1.91 (1.72–2.12)***	1.51 (1.34–1.70)***

Significant values are in bold.

*SEIFA* Socio-Economic Indexes for Areas, *IRSAD* Index of Relative Socio-economic Advantage and Disadvantage, with quintile 5 indicating the highest levels of socio-economic advantage and quintile 1 the lowest levels of socio-economic advantage. *p* values included where significant *p < 0.05; **p < 0.01; ***p < 0.001, ^a^n = 10,766. Significant results are represented in black font. All variables included in the model. ^b^missing data from ‘don’t know’, ‘refused’ or ‘unable to determine value’. ^c^Other includes home duties, non-working students and other (unspecified).

### Ethical approval

No ethics approval and consent were required for this study. This project was granted exemption from ethics review from The University of Sydney Human Research Ethics Committee.

## Results

### Prevalence of subtypes of loneliness and social isolation

**Figure 1:**
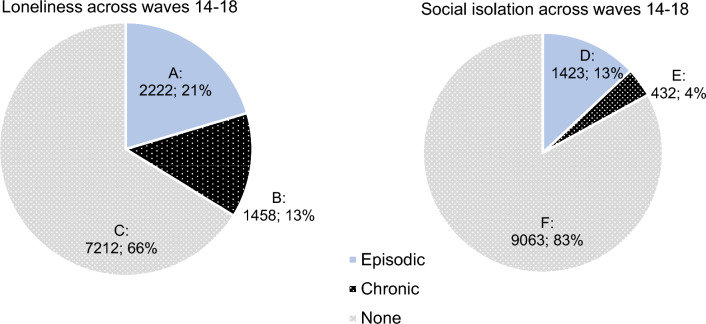
Prevalence of (**A**) episodic loneliness, (**B**) chronic loneliness, (**C**) no loneliness; (**D**) episodic social isolation, (**E**) chronic social isolation, and (**F**) no social isolation across 5 years.

At least 34% of participants reported either episodic or chronic loneliness during waves 14 (2014) to 18 (2018). Of these, 21% experienced an episode of loneliness and 13% experienced chronic loneliness. The prevalence of social isolation was lower than that of loneliness. Only 17% of participants reported either episodic or chronic social isolation during waves 14 (2014) to 18 (2018). Of this, 13% reported episodic social isolation, and 4% reported chronic social isolation (See Fig. 1).

### Correlates of subtypes of loneliness and social isolation

Findings from the analysis of the relationship between socio-demographic and health factors and the dependent variables of loneliness and social isolation are shown in Tables 1 and 2, respectively.

#### Sex

Being male was associated with lower likelihood of reporting loneliness (episodic or chronic) compared to females by 11% (OR 0.89, 95% CI 0.82–0.96), but these relationships became insignificant once adjusted for all covariates. Conversely, being male was associated with a higher likelihood of both episodic (AOR 1.23, 95% CI 1.09–1.38) and chronic social isolation (AOR 1.78; 95%CI 1.45–2.20).

#### Age

In order to examine differences across age groups, we used 30–44 years as the reference category given that previous studies have reported this group as being less lonely than the younger age group (15–29 years) and we wished to determine the magnitude of any increased risk in the youngest group. When compared to those aged 30–44 years, younger people aged 15–29 were less likely to report chronic loneliness (AOR 0.77, 95% CI 0.62–0.94). However, those aged 60–74 years and those 75 + also showed the same trend reporting significantly less chronic loneliness than those aged 30–44 years. (60–74: AOR 0.62, 95% CI 0.49–0.78; 75 +: AOR 0.59 95% CI 0.43–0.81). Those aged 45–49 were also less likely to experience either subtype of loneliness (AOR 0.85, 95% CI 0.75–0.97). In regard to social isolation*,* only the 45–59 age group reported significantly more of either subtype of social isolation than those aged 30–44 years (AOR 1.19 95% CI 1.01–1.40).

#### Household structure

When compared with couples with children, living alone (AOR 1.66, 95% CI 1.36–2.03) and being a lone parent with a child increased the likelihood of chronic loneliness (AOR 1.64, 95% CI 1.19–2.27). Couples without children were significantly less likely to experience chronic loneliness (AOR 0.75, 95% CI 0.62–0.92) when compared with couples with children. Similarly, when compared with couples with children, people who lived alone or in other household types were more likely to be episodically or chronically social isolated. Lone parents with children were more likely to be episodically socially isolated compared to couples with children (AOR 1.50, 95% CI 1.05–2.15).

### Country of birth and language other than English

Those born in a country other than Australia (non-English speaking) are more likely to report episodic loneliness than those born in Australia (AOR 1.22, 95% CI 1.01–1.48), but were significantly less likely to report chronic loneliness (AOR 0.83, 95% CI 0.69–1.00). There were no differences in levels of loneliness between people who spoke a language other than English and people who spoke English. People born outside of Australia both in English and non-English speaking countries did not report an increased likelihood of social isolation compared with those born in Australia. Compared with people who spoke only English, those who spoke a language other than English did not show higher levels of social isolation after results were fully adjusted (all *ps* > 0.05).

#### Household income

Compared to participants who reported the highest income (over $150,000), participants who reported the lowest income (< $80,000) and those who reported 80,000–149,000 had an increased likelihood of both episodic (< $80,000: AOR 1.49, 95% CI 1.27–1.74; $80,000–149,000: AOR 1.27 95% CI 1.10–1.45) and chronic loneliness (< $80,000: AOR 1.66, 95% CI 1.36–2.02; $80,000–149,000: AOR 1.26 95% CI 1.05–1.50). In addition, participants who reported the lowest income (< $80,000) had an increased likelihood of both episodic (AOR 1.48, 95% CI 1.22–1.80) and chronic social isolation (AOR 1.83, 95% CI 1.30–2.57). Those who those earned between $80,0000 to $149,999, showed an increased likelihood of episodic social isolation (AOR 1.23, 95% CI 1.04–1.47) when compared to those who earned over $150,000, but not chronic social isolation.

#### Employment status

When compared with those in full-time employment, those who were unemployed and those who in ‘other’ category of employment (i.e., home duties, non-working students) were more likely to report episodic (unemployed: AOR 1.33, 95% CI 1.01–1.77; other: AOR 1.21, 95% CI 1.02–1.77) and chronic loneliness (unemployed: AOR 1.66, 95% CI 1.22–2.25; other: AOR 1.57, 95% CI 1.29–1.91). Those who were unemployed and those who in ‘other’ category were also more likely than those in full-time employment to report episodic (unemployed: AOR 1.43, 95% CI 1.03–1.97; other: AOR 1.47, 95% CI 1.19–1.80) and chronic social isolation (unemployed: AOR 2.46, 95% CI 1.88–3.24; other: AOR 2.21, 95% CI 1.88–2.60).

#### SEIFA IRSAD

Compared with participants in SEIFA IRSAD 5 (most advantaged), only participants in SEIFA IRSAD quintiles 1 (least advantaged) showed an increased likelihood of both episodic (AOR 1.36 95% CI 1.16–1.59) and chronic loneliness (AOR 1.34 95% CI 1.11–1.62). On the other hand, participants in SEIFA IRSAD quintiles 1 to 2 had an increased likelihood of both episodic social isolation (quintile 1: AOR 1.63, 95% CI 1.35–1.97; quintile 2: AOR 1.24, 95% CI 1.03–1.50) and chronic social isolation (quintile 1: AOR 1.69, 95% CI 1.15–2.48, quintile 2: AOR 1.77, 95% CI 1.21–2.59). Those in quintiles 3 and 4 had an increased likelihood of chronic social isolation (quintile 3: AOR 2.33, 95% CI 1.61–3.38; quintile 4: AOR 2.11, 95% CI 1.46–3.06) compared with those in quintile 5.

### Long-term health condition

Participants who reported a long-term health condition showed an increased likelihood of both episodic (AOR 1.24, 95% CI 1.11–1.39) and chronic loneliness (AOR 2.01, 95% CI 1.76–2.29) than those who did not. In addition, participants who reported a long-term health condition reported an increased likelihood of both episodic (AOR 1.34, 95% CI 1.17–1.52), and chronic social isolation (AOR 1.87, 95% CI 1.50–2.34).

## Discussion

Loneliness and social isolation have been recognised as important public health issues in many countries. While these conditions have similar negative health impacts, it is unclear *who* is more likely to experience chronic loneliness and social isolation. This study is the first to distinguish episodic from chronic experiences of loneliness and social isolation with the use of longitudinal population data, and to identify who is most vulnerable.

It was noteworthy that the cumulative prevalence rates of loneliness (overall 34%; 21% episodic, 13% chronic) far exceeded the prevalence rates of social isolation (overall 17%; 13% episodic, 4% chronic) in Australia. It is also possible that the cumulative prevalence rate of loneliness in this study is underestimated, given that the HILDA participants themselves may be more engaged or less socially isolated than the general Australian community. However, more programs and policies have focussed on reducing social isolation and fewer on reducing loneliness—perhaps simply because it is easier to measure a reduction in social isolation (i.e., objective, quantifiable, and or observable) as opposed to the challenges of measuring loneliness (i.e., subjective, qualitative). For example, employing strategies that increase social contact and opportunities (i.e., reduce social isolation) may not also lead to the development and maintenance of meaningful connections, which could reduce loneliness. Several factors may be driving this, including a gap in research translation to practice (e.g., community and health practitioners not measuring loneliness severity) to poor community awareness about what loneliness is (e.g., confusion with social isolation or stigma associated with loneliness)^58^.

Being male was protective of any type (episodic or chronic) of loneliness, consistent with previous study which report women are more predisposed to loneliness^59^, including older women^60^. Men, however, were more likely to be socially isolated than women, which is consistent with sex differences in reporting subjective versus objective social isolation^61,62^. Although men were more socially isolated, they may be either less vulnerable or more reluctant to report loneliness.

Our results indicate that age differences were only present in those that reported chronic, as opposed to episodic, loneliness. Much of the research to date does not make the distinction between these sub-types, but most point to a U-shaped distribution where younger and older people are more vulnerable to problematic levels of loneliness^16,49^. After accounting for all possible confounding variables, those aged 75 years and older, followed by those aged 60–74 years, and those 15–29 years were all less likely to experience chronic loneliness than those aged 30–44 years. One plausible reason for the greater vulnerability of individuals aged 30–44 years could include a lack of time to nurture and maintain meaningful social relationships.

Interestingly, the only age-related difference in social isolation was the higher prevalence among those aged 45–59 years of either subtype. While there is a strong focus upon social isolation in older adults in both research and public policy, our findings indicate there is a need to assist individuals in the middle-aged, pre-retirement phase of life. Those aged 45–59 years were also less likely to experience any episodic or chronic loneliness, which demonstrates that these constructs are likely to be independent.

Overall, these findings challenge previous studies about who is lonely and who is socially isolated. These differing results are likely to be due to the heterogeneity of samples and different measures of loneliness and social isolation used, and the inclusion of confounding factors (e.g. household structure) are often not included in other studies. Further, our psychometrically derived scales for loneliness and social isolation may be robust but is only specifically derived from the HILDA^44^. This makes it difficult to directly compare with other studies and more research is needed to understand the specific factors that may drive social vulnerability in particular groups.

Our findings indicate that single parents with young children have an elevated risk of chronic loneliness, almost equivalent to those who live alone. Similarly, an earlier study using the HILDA sample also indicate that single fathers with children were at risk of loneliness^43^. While there no known studies examining the impact of loneliness in single parents, the detrimental impact is consistent with reports of poor health status^63^ and increased mortality risk in this population group^64^. It was noteworthy that couples without children were less likely to experience chronic loneliness when compared with couples with children. Further, compared with those without children, almost all groups reported higher levels of episodic social isolation but only those who lived alone and who lived with non-family members were more likely to experience chronic social isolation.

We found no differences between by country of birth (Australia, other English speaking, or non-English speaking) for episodic or chronic social isolation. On the other hand, people from non-English speaking countries were at more risk of episodic loneliness, while tending to have lower risk of chronic loneliness. This may signal that individuals from non-English speaking backgrounds are able to build meaningful social relationships as their duration of residence lengthens. Further research needs to be done comparing loneliness and social isolation between specific cultural groups with appropriate assessment tools, especially in light of research showing that loneliness is a significant predictor of lower self-reported health, greater risk of posttraumatic stress, and higher incidence of mental illness in migrant groups^65^.

Household income was found to have an inverse relationship with the risk of both episodic and chronic loneliness, that is, the lower the income the higher likelihood of reporting each sub-type of loneliness. Similar patterns were found for episodic social isolation, but only those who had the lowest income (< $80,000) reported higher likelihood of chronic social isolation. Plausible reasons for these trends include having fewer resources (i.e., time or money) to invest in developing and maintaining meaningful social connection (i.e., reducing their risk of loneliness and social isolation)^61^. It was also found that those who were unemployed showed the highest risk of both episodic and chronic loneliness, but compared with those in full-time employment, people engaged in home duties and non-working students showed the highest risk of episodic and chronic social isolation. This highlights the opportunities that employment can offer for building and developing social connection.

Consistent with previous research^66–68^, those living in more disadvantaged neighbourhoods reported more loneliness and social isolation compared with people living in more advantaged neighbourhoods. More advantaged neighbourhoods may offer more physical spaces and environmental resources (such as green spaces) that can be conducive to promoting social connection^69^. A similar clear trend was seen for episodic social isolation, with those in the most more disadvantaged neighbourhoods (SEIFA IRSAD 1–2) having the highest risk of episodic social isolation, whereas all groups (SEIFA IRSAD 1–4) were at greater risk of chronic social isolation than those on the most advantaged quintile (SEIFA IRSAD 5).

People who had a long-term health condition were consistently more likely to experience both loneliness and socially isolation, with this sub-group reporting approximately double the risk of chronic loneliness and isolation compared to those without a long-term health condition. Approximately, one in four (24%) individuals with a long-term health condition reported episodic loneliness and one in five (20%) met the criteria for chronic loneliness. While there is a plethora of research on how individual health characteristics (i.e., physical health, BMI)^70,71^, and poor health regulation behaviours (i.e., smoking, alcohol use, physical activity)^72,73^ influence loneliness and social isolation in specific demographic cohorts, there is a lack of clarity on how these factors influence the onset of chronic loneliness. Our findings nevertheless highlight the importance of preventing loneliness in these vulnerable groups, and the need to equip health care practitioners and community agencies to better support people with long-term health conditions to manage their psychosocial well-being^74^. This may be in the form of building linkages with others who have a shared experience and to facilitate greater participation with their existing social networks.

## Limitations

While the sample used in analysis was weighted to match the profile of the Australian population, it is possible that individuals taking part in the HILDA longitudinal study are unrepresentative in selected characteristics (social and psychological) that are of importance to this study and cannot be mitigated by means of demographic weighting. Further, these issues may be magnified by the fact participants under 18 were primarily recruited via their parents, who are themselves HILDA participants and this recruitment method may skew our results on loneliness and social isolation reported by young people under 18.

This study enabled examination of how loneliness and social isolation of differing levels of duration affect particular population groups, using psychometrically validated scales but the categorical classification did not enable investigation of the severity (i.e., intensity) of these experiences. The differences between episodic and chronicity of these social experiences is at its infancy. One outstanding question is whether the negative impact of an intense but short episodic of loneliness and social isolation is equivalent to the negative impact of a low intensity but persistent experience of loneliness and social isolation. Additionally, this study does not show the factors predicting a transition from episodic loneliness and social isolation to the chronic forms of these conditions, which remains an evidence-gap^75^.

This study did not examine more closely differences in how loneliness and social isolation influence different chronic health conditions^76^, as there is evidence that the pathway to disease could differ for loneliness and social isolation (e.g., loneliness better predicted poorer mental health; social isolation better predicted poorer physical and cognitive health)^77^, Previous relationships have been established between specific health conditions and loneliness and social isolation, for example, cardiovascular disease and Type 2 diabetes were associated with loneliness and social isolation, but the same effect was not found for other disorders including chronic obstructive pulmonary disease and cancer^28^.

## Implications

This study provides an indication of who is vulnerable to loneliness and social isolation, especially within the Australian context. Our data shows clearly that economic and social factors influence our social connection—for example, people with low income, who are unemployed and live in disadvantaged neighbourhoods are at greater risk of loneliness and social isolation. Hence, these conditions are inherently linked with fundamental social determinants of health^15^.

The outstanding question is how can we effectively address loneliness and social isolation, and improve social connection in the long term? Many solutions adopt a downstream approach, to help people who are already lonely to manage their distressing feelings. This may involve delivering individually based (e.g., therapy) or community-based programs (e.g., improving neighbourhood connections). However, these solutions may offer only short term benefits if we do not consider a whole-of-systems approach to loneliness and social isolation.

There must be continued efforts of leveraging cross-sector collaborations (e.g., health, business, and community sectors) to also take upstream solutions (e.g., improving access to affordable housing, employment support). These upstream solutions offer high potential for preventing the onset of loneliness and social isolation and yield long-term benefits^78^.

## Conclusion

Having social connection, be it having more contact with others or feeling meaningfully connected to them, is fundamental for health and wellbeing. Our findings indicate that loneliness when compared with social isolation both in episodic and chronic subtypes, is more prevalent than social isolation. Both chronic forms loneliness and social isolation remains neglected and poorly targeted within current practice and policy. Further, we have found that individuals who are socially disadvantaged and those with long-term health conditions are more likely to report episodic and chronic loneliness and social isolation. This highlights the need to better understand the psychological, economic, and environmental mechanisms that are contributing to loneliness and social isolation in these population groups, and to use this knowledge to develop policies and programs that address these critical dimensions of health and quality of life.

## Data Availability

The dataset supporting the conclusions of this article are available to researchers living in Australia or overseas through the National Centre for Longitudinal Data Dataverse. Information about applying for access to the data is available at: https://dataverse.ada.edu.au/dataverse/ncld. This study was exempted from ethics review from The University of Sydney Human Research Ethics Committee.

## References

[1] Cacioppo JT, Cacioppo S (2014). Social relationships and health: The toxic effects of perceived social isolation. Soc. Pers. Psychol. Compass.

[2] Coyle CE, Dugan E (2012). Social isolation, loneliness and health among older adults. J. Aging Health.

[3] Victor C, Scambler S, Bond J, Bowling A (2000). Being alone in later life: Loneliness, social isolation and living alone. Rev. Clin. Geront..

[4] Steptoe A, Shankar A, Demakakos P, Wardle J (2013). Social isolation, loneliness, and all-cause mortality in older men and women. PNAS.

[5] Pantell M (2013). Social isolation: A predictor of mortality comparable to traditional clinical risk factors. Am. J. Public Health.

[6] Cacioppo JT, Cacioppo S, Capitanio JP, Cole SW (2015). The neuroendocrinology of social isolation. Annu. Rev. Psychol..

[7] Peplau L, Perlman D (1982). Loneliness: A Sourcebook of Current Theory, Research and Therapy.

[8] Petitte T (2015). A systematic review of loneliness and common chronic physical conditions in adults. Open Psychol. J..

[9] Cacioppo S, Capitanio JP, Cacioppo JT (2014). Toward a neurology of loneliness. Psychol. Bull..

[10] Valtorta NK, Kanaan M, Gilbody S, Ronzi S, Hanratty B (2016). Loneliness and social isolation as risk factors for coronary heart disease and stroke: Systematic review and meta-analysis of longitudinal observational studies. Heart.

[11] Kuiper JS (2015). Social relationships and risk of dementia: A systematic review and meta-analysis of longitudinal cohort studies. Ageing Res. Rev..

[12] Lim MH, Rodebaugh TL, Zyphur MJ, Gleeson JF (2016). Loneliness over time: The crucial role of social anxiety. J Ab Psychol.

[13] Holt-Lunstad J, Smith TB, Baker M, Harris T, Stephenson D (2015). Loneliness and social isolation as risk factors for mortality: A meta-analytic review. Pers. Psychol. Sci..

[14] Henriksen J, Larsen ER, Mattisson C, Andersson NW (2019). Loneliness, health and mortality. Epidemiol. Psychiatr. Sci..

[15] Lim MH, Eres R, Vasan S (2020). Understanding loneliness in the twenty-first century: An update on correlates, risk factors, and potential solutions. Soc. Psychiatry Psychiatr. Epidemiol..

[16] Victor CR, Yang K (2012). The prevalence of loneliness among adults: A case study of the United Kingdom. J Psychol.

[17] Qualter P (2015). Loneliness across the lifespan. Pers. Psych. Sci..

[18] Spruce L (2019). Back to basics: social determinants of health. Aorn. J..

[19] Coughlin SS, Young L (2020). Social determinants of myocardial infarction risk and survival: A systematic review. Eur. J. Cardiovasc. Res..

[20] Coughlin SS (2020). Social determinants of colorectal cancer risk, stage, and survival: A systematic review. Int. J. Colorectal Dis..

[21] Climie RE (2019). Individual and neighborhood deprivation and carotid stiffness. Hypertension.

[22] Kim ES, Chen Y, Kawachi I, Vander Weele TJ (2020). Perceived neighborhood social cohesion and subsequent health and well-being in older adults: An outcome-wide longitudinal approach. Health Place.

[23] Mann F (2017). A life less lonely: The state of the art in interventions to reduce loneliness in people with mental health problems. Soc. Psychiatry Psychiatr. Epidemiol..

[24] Cacioppo JT, Hawkley LC (2009). Perceived social isolation and cognition. Trends Cogn. Sci..

[25] Cacioppo JT, Cacioppo S, Boomsma DI (2014). Evolutionary mechanisms for loneliness. Cogn. Emot..

[26] Lim MH, Gleeson JFM, Alvarez-Jimenez M, Penn DL (2018). Loneliness in psychosis: A systematic review. Soc. Psychiatry Psychiatr. Epidemiol..

[27] Theeke L (2010). Sociodemographic and health-related risks for loneliness and outcome differences by loneliness status in a sample of US older adults. Res. Gerontol. Nurs..

[28] Christiansen J (2020). Loneliness, Social isolation, and chronic disease outcomes. Ann. Behav. Med..

[29] Surkalim DL (2022). The prevalence of loneliness across 113 countries: systematic review and meta-analysis. BMJ.

[30] Caspi A, Harrington H, Moffitt TE, Milne BJ, Poulton R (2006). Socially isolated children 20 years later: risk of cardiovascular disease. Arch. Pediatr. Adoles. Med..

[31] Mund M, Freuding MM, Möbius K, Horn N, Neyer FJ (2020). The stability and change of loneliness across the life span: A meta-analysis of longitudinal studies. Pers. Soc. Psychol. Rev..

[32] Office for National Statistics. *What Characteristics and Circumstances are Associated with Feeling Lonely*? https://www.ons.gov.uk/peoplepopulationandcommunity/wellbeing/articles/lonelinesswhatcharacteristicsandcircumstancesareassociatedwithfeelinglonely/2018-04-10 (2018).

[33] Russell DW (1996). UCLA loneliness scale (version 3): Reliability, validity, and factor structure. J. Pers. Assess..

[34] De Jong Gierveld J, van Tilburg T (2008). A shortened scale for overall, emotional and social loneliness. Tijdschr. Gerontol. Geriatr..

[35] Martín-María N (2020). Differential impact of transient and chronic loneliness on health status. A longitudinal study. Psychol. Health.

[36] Newall NE, Chipperfield JG, Bailis DS (2014). Predicting stability and change in loneliness in later life. J. Soc. Pers. Relat..

[37] Shiovitz-Ezra S, Ayalon L (2010). Situational versus chronic loneliness as risk factors for all-cause mortality. Int. Psychogeriatr..

[38] Zhong B-L, Chen S-L, Conwell Y (2016). Effects of transient versus chronic loneliness on cognitive function in older adults: Findings from the Chinese Longitudinal Healthy Longevity Survey. Am. J. Geriatr. Psychiatry.

[39] Matthews T (2022). The developmental course of loneliness in adolescence: Implications for mental health, educational attainment, and psychosocial functioning. Dev. Psychopathol..

[40] Wilkins R, Laß I, Butterworth P, Vera-Toscano E (2019). The Household, Income and Labour Dynamics in Australia Survey: Selected Findings from Waves 1 to 17.

[41] Butterworth P, Crosier T (2004). The validity of the SF-36 in an Australian National Household Survey: Demonstrating the applicability of the Household Income and Labour Dynamics in Australia (HILDA) Survey to examination of health inequalities. BMC Public Health.

[42] Lau S, Gruen GE (1992). The social stigma of loneliness: Effect of target person's and perceiver's sex. Pers. Soc. Psychol. Bull..

[43] Flood, M. *Mapping Loneliness in Australia. The Australia Institute.* https://australiainstitute.org.au/wp-content/uploads/2020/12/DP76_8.pdf (2005)

[44] Manera KE, Smith BJ, Owen KB, Phongsavan P, Lim MH (2022). Psychometric assessment of scales for measuring loneliness and social isolation: An analysis of the household, income and labour dynamics in Australia (HILDA) survey. Health Qual. Life Outcomes.

[45] National Centre for Chronic Disease Prevention and Health Promotion (NCCDPHP). *About Chronic Disease*. https://www.cdc.gov/chronicdisease/about/index.htm (2021).

[46] Umberson D, Lin Z, Cha H (2022). Gender and social isolation across the life course. J. Health Soc. Behav..

[47] Theeke LA (2009). Predictors of loneliness in U.S. adults over age sixty-five. Arch. Psychiatr. Nurs..

[48] Tong S (2019). Geographic characteristics of loneliness in primary care. Ann. Fam. Med..

[49] Lasgaard M, Friis K, Shevlin M (2016). “Where are all the lonely people?” A population-based study of high-risk groups across the life span. Soc. Psychiatry Psychiatr. Epidemiol..

[50] Lee SB, Shin Y, Jeon Y, Kim S (2022). Factors affecting social isolation among the young adults in South Korea: A cross-sectional analysis. Front. Pub. Health.

[51] Becker CC (2022). Migrants’ social integration and its relevance for national identification: An empirical comparison across three social spheres. Front. Sociol..

[52] Sawir E, Marginson S, Deumert A, Nyland C, Ramia G (2008). Loneliness and international students: An Australian study. J. Stud. Int. Educ..

[53] Zhao IY, Holroyd E, Garrett N, Wright-StClair VA, Neville S (2023). Chinese late-life immigrants' loneliness and social isolation in host countries: An integrative review. J. Clin. Nurs..

[54] Algren MH (2020). Social isolation, loneliness, socioeconomic status, and health-risk behaviour in deprived neighbourhoods in Denmark: A cross-sectional study. SSM Popul. Health.

[55] Australian Bureau of Statistics. *Census of Population and Housing: Reflecting Australia: Stories from the Census, 2016.* https://www.abs.gov.au/ausstats/abs@.nsf/Lookup/by%20Subject/2071.0~2016~Main%20Features~Socio-Economic%20Advantage%20and%20Disadvantage~123 (2016).

[56] Crowe CL (2021). Associations of loneliness and social isolation with health span and life span in the U.S. Health and Retirement study. J. Geront. Ser..

[57] Summerfield M (2022). HILDA User Manual-Release 21.

[58] Ending Loneliness Together. *Ending Loneliness Together in Australia Whitepaper*. https://endingloneliness.com.au/wp-content/uploads/2020/11/Ending-Loneliness-Together-in-Australia_Nov20.pdf (2020).

[59] Borys S, Perlman D (1985). Gender differences in loneliness. Pers. Soc. Psychol. Bull..

[60] Dahlberg L, Andersson L, McKee KJ, Lennartsson C (2015). Predictors of loneliness among older women and men in Sweden: A national longitudinal study. Aging Ment. Health.

[61] Pinquart M, Sorensen S (2001). Influences on loneliness in older adults: A meta-analysis. Basic Appl. Soc. Psychol..

[62] Pinquart M (2003). Loneliness in married, widowed, divorced, and never-married older adults. J. Soc. Pers. Relat..

[63] Chiu M (2017). Self-rated health and mental health of lone fathers compared with lone mothers and partnered fathers: A population-based cross-sectional study. J. Epidemiol. Commun. Health.

[64] Chiu M (2018). Mortality in single fathers compared with single mothers and partnered parents: A population-based cohort study. Lancet Public. Health.

[65] Chen W, Wu S, Ling L, Renzaho AMN (2019). Impacts of social integration and loneliness on mental health of humanitarian migrants in Australia: Evidence from a longitudinal study. Aust. N. Z. J. Public Health.

[66] Creed PA, Reynolds J (2001). Economic deprivation, experiential deprivation and social loneliness in unemployed and employed youth. J. Commun. Appl. Soc. Psychol..

[67] Kearns A, Whitley E, Tannahill C, Ellaway A (2014). Loneliness, social relations and health and well-being in deprived communities. Psychol. Health Med..

[68] McCain D, Morgan AA, Wright R (2021). Associations between neighborhood SES disadvantage and feelings of depression and loneliness in older adults. Innov. Aging.

[69] Astell-Burt T (2021). More green, less lonely? A longitudinal cohort study. Int. J. Epidemiol..

[70] Christiansen J (2021). Associations of loneliness and social isolation with physical and mental health among adolescents and young adults. Pers. Public. Health.

[71] Fernández-Alonso AM, Trabalón-Pastor M, Vara C, Chedraui P, Pérez-López FR (2012). Life satisfaction, loneliness and related factors during female midlife. Maturitas.

[72] Shankar A, McMunn A, Banks J, Steptoe A (2011). Loneliness, social isolation, and behavioral and biological health indicators in older adults. Health Psychol..

[73] Schrempft S, Jackowska M, Hamer M, Steptoe A (2019). Associations between social isolation, loneliness, and objective physical activity in older men and women. BMC Public Health.

[74] Mutz J, Roscoe CJ, Lewis CM (2021). Exploring health in the UK Biobank: Associations with sociodemographic characteristics, psychosocial factors, lifestyle and environmental exposures. BMC Med..

[75] Qualter, P. *et al. Tackling Loneliness Evidence Review: Main Report*. https://www.gov.uk/government/publications/tackling-loneliness-evidence-review/tackling-loneliness-evidence-review-full-report (2022).

[76] Stickley A, Koyanagi A (2018). Physical multimorbidity and loneliness: a population-based study. PLoS ONE.

[77] Beller J, Wagner A (2017). Disentangling loneliness: Differential effects of subjective loneliness, network quality, network size, and living alone on physical, mental, and cognitive health. J. Aging Health.

[78] Stansfield J, South J, Mapplethorpe T (2020). What are the elements of a whole system approach to community-centred public health? A qualitative study with public health leaders in England’s local authority areas. BMJ Open.

